# COVID-19 Pathogenesis: From Molecular Pathway to Vaccine Administration

**DOI:** 10.3390/biomedicines9080903

**Published:** 2021-07-27

**Authors:** Francesco Nappi, Adelaide Iervolino, Sanjeet Singh Avtaar Singh

**Affiliations:** 1 Department of Cardiac Surgery, Centre Cardiologique du Nord, 93200 Saint-Denis, France; 2 Department of Cardiovascular Sciences, Fondazione Policlinico Universitario Agostino Gemelli IRCSS, 00168 Rome, Italy; adelaide.iervolino@libero.it; 3 Department of Cardiothoracic Surgery, Golden Jubilee National Hospital, Glasgow G81 4DY, UK; sanjeetsinghtoor@gmail.com

**Keywords:** SARS-CoV-2, COVID-19, thromboembolism, ACE inhibition, pathophysiology

## Abstract

The Coronavirus 2 (SARS-CoV-2) infection is a global pandemic that has affected millions of people worldwide. The advent of vaccines has permitted some restitution. Aside from the respiratory complications of the infection, there is also a thrombotic risk attributed to both the disease and the vaccine. There are no reliable data for the risk of thromboembolism in SARS-CoV-2 infection in patients managed out of the hospital setting. A literature review was performed to identify the pathophysiological mechanism of thrombosis from the SARS-CoV-2 infection including the role of Angiotensin-Converting Enzyme receptors. The impact of the vaccine and likely mechanisms of thrombosis following vaccination were also clarified. Finally, the utility of the vaccines available against the multiple variants is also highlighted. The systemic response to SARS-CoV-2 infection is still relatively poorly understood, but several risk factors have been identified. The roll-out of the vaccines worldwide has also allowed the lifting of lockdown measures and a reduction in the spread of the disease. The experience of the SARS-CoV-2 infection, however, has highlighted the crucial role of epidemiological research and the need for ongoing studies within this field.

## 1. Introduction

Patients with severe acute respiratory syndrome Coronavirus 2 (SARS-CoV-2) infection may develop associated arterial and venous thrombotic complications. Data reported in the 2019 U.S. Coronavirus Disease Patient Registry (COVID-19) recorded a 2.6% rate of thrombotic complications in the 299 patients who required non-critical hospitalization compared to the rate of 35.3% of the 170 patients hospitalized in critical care units [1,2]. Klok et al. confirmed a remarkably high 31% incidence of thrombotic complications in ICU patients with COVID-19 infections^2^. These results supported the recommendation to use drug prophylaxis for thrombosis in all COVID-19 patients admitted to the ICU. The data strongly support increased drug prophylaxis dosage, even in the absence of randomized trials.

To date, there are no reliable data to establish the risk of thromboembolism in SARS-CoV-2 infection in patients whose clinical conditions do not require hospitalization. Several studies reported that patients admitted to the hospital with COVID-19 disease experienced thrombotic complications involving the heart, brain, and peripheral vascular system, which mainly led to myocardial infarction (MI), ischemic stroke, and venous thromboembolism (VTE) [3,4,5].

During the initial months of the pandemic acceleration, several autopsy studies [6,7] highlighted the presence of systemic microthrombi in many organs, including lungs, heart, and kidneys, thereby suggesting how thrombosis could contribute to the frequent and often fatal multisystem organ failure in patients with severe COVID-19 disease [8,9].

### 1.1. Angiotensin-Converting Enzyme (ACE) 2 Receptor: The Evolutionary Stage of Infection from Himalayan Palm Civet and Bat Coronavirus to SARS-CoV2 Infection

The gateway for SARS-CoV-2 to target cells is the angiotensin-converting enzyme (ACE) 2 receptor, which is mostly expressed by epithelial cells of the lung, heart, blood vessels, kidneys, and intestines. The ACE family of receptors includes both ACE and ACE2 which, although they both are dipeptidyl mono-carboxydipeptidases have distinct physiological functions.

### 1.2. Structure of the ACE as Ligand-Binding Receptors

SARS-CoV-2 uses common cellular transmission which is based on the binding of ligands to specific cell surface receptors. ACE2 is a G protein-coupled receptor (GPCR) and belongs to a category of receptors that play a central role in the initiation and regulation of cellular processes [10]. The GPCR constitutes the most prominent class of receptors implicated in pathological disorders of the cardiovascular, respiratory, endocrine, immune, and neural systems. Activation of GPCRs is also common in neoplastic pathologies. The function that GPCRs exert is mediated by responses to specific interactions with hormones, neurotransmitters, pathogens, metabolites, ions, fatty acids, and drugs [11,12].

GPCRs are crucial modulators of transmission between the internal and external environment of cells. GPCRs are integral membrane proteins with an extracellular N-terminal and seven transmembrane (TM) helical domains, from TM1 to TM7, connected via link regions. Evidence suggested that GPCRs have a more complex role than originally considered. The binding of GPCRs to very different types of extracellular stimuli leads to conformational changes of the TM domain with the consequent structural remodeling of the protein [13,14,15,16,17]. Inter alia, these conformation changes induce coupling with cytoplasmic proteins and subsequently the activation of enzymes that lead to the generation of a second messenger. Once the second messenger is formed it can activate a sequence of signals inside the cell [14]. This specific role of GPCRs results in increasing levels of intracellular cyclic Adenosine Monophosphate (cAMP) and represents the pivotal pathway in response to ligands, such as signaling of the renin–angiotensin system (RAS). It is important to underline that the levels of cAMP production in the cellular domain are modulated by several factors. Multidrug Resistance Proteins (MRPs) allow the efflux of cAMP from the inside of the cell to the extracellular fluid, thus maintaining homeostatic intracellular concentrations. The role of transporters activated by MRPs serves to regulate the balance of cAMP within the cell.

Lu et al. [15] reported concern about the structural conformation of the ACE/GPCR complex and its interaction with SARS-CoV by focusing on lipid rafts. The structure, activation, and signaling of the ACE/GPCR complex are strongly influenced by the bilayer domain with specific membrane-GPCR interactions [16]. It has been shown that some subsets of GPCR are preferentially isolated in distinct regions of the membrane defined as lipid rafts [17,18,19]. Cholesterol partitions preferentially into lipid rafts which contain 3 to 5-fold the amount of cholesterol found in the surrounding bilayer. Evidence has shown that lipid rafts serve as an entry site for SARS-CoV. For example, lipid rafts in Vero E6 cells were involved in the “entry” of the coronavirus of the severe acute respiratory syndrome (SARS-CoV). As has been clarified by the tests after SARS-CoV infection, the integrity of the lipid rafts was a necessary requirement to produce the pseudotyped SARS-CoV infection. If plasma membrane cholesterol depletion was induced using the relocalized MbetaCD marker on the caveolin raft the SARS-CoV, ACE2 receptor was not significantly modified. Although the surface expression of ACE2 still allowed binding to the virus, treatment with MbetaCD inhibited the infectivity of the pseudotyped SARS-CoV by 90%. The observed data concern the ectodomain of the SARS-CoV protein S (S1188HA) which can be associated with lipid rafts. The spike protein, after binding to its receptor, colocalized with the ganglioside marker GM1 residing on the raft. The study found that S1188HA binding was not affected by plasma membrane cholesterol depletion supporting the conclusion that lipid rafts serve as a gateway for SARS-CoV [20,21,22,23,24].

### 1.3. Function of ACE Receptor

The function of ACE is to split angiotensin I into angiotensin II, which binds and activates the type 1 angiotensin II receptor. This activation triggers a series of pathophysiological mechanisms that ultimately have vasoconstrictor, proinflammatory, and pro-oxidative effects. It is important to underline that among the functions of ACE2 is the hydrolytic degradation of angiotensin II to angiotensin 1–7 and angiotensin I to angiotensin 1–9. Once angiotensin 1–9 is generated, it binds to the Mas receptor, producing anti-inflammatory, antioxidant, and vasodilatory reactions. From a pathophysiological point of view, it is important to distinguish the two forms of ACE2 receptors. The first is a type 1 integral transmembrane protein with structural features representing the extracellular domain that acts as a receptor for the SARS-CoV-2 spike protein. The second is a soluble form representing circulating ACE2. To date, our knowledge is limited on the relationship that is established between SARS-CoV-2 and the two forms of the receptor. A better understanding of this relationship may more precisely define the operational adaptive or maladaptive processes that sustained COVID-19 infection [25,26].

### 1.4. ACE Receptor and Binding to Human Coronary Viruses

The knowledge we have on the interaction between the human ACE2 receptor (hACE2) and the Himalayan palm civet receptor (cACE2) with SARS-CoV derives from the usage of the receptor by the Human (hSARS-CoV) and Himalayan palm civet coronavirus (cSARS-CoV) [27]. The hSARS- CoV can bind both hACE2 and cACE2 receptors while the palm civet coronavirus has no interaction with the ACE2 receptor expressed in humans. It is known that the adaptation of c SARS-CoV to humans was determined by two-point mutations, recognized as K479N and S487T, in the binding domain of the SARS-CoV spike protein (SARS-CoV-S) [26].

The mutations that have recently characterized SARS-CoV-2 led to more aggressive variants of the virus and the concept of adaptive mutations (as noted by Wu et al. [28]) with strengthened receptor binding and tropism (RBT). The authors demonstrated that adaptive mutations of RBT led to the identification of genetic mutations of the virus that enhanced interaction with human or palm civet ACE2. The genetic adaptation processes that took place between hSARS-CoV and cSARS-CoV could also be recorded in SARS-CoV-like viruses that have been isolated in bats [28]. A previous study found that the pathways in which bat coronaviruses infected host cells did not occur through the interaction of the ACE2 receptor with expressed SARS-CoVS and remained a mystery. However, the important finding remains that the substitution of the amino acid sequence found between residues 323 and 505 of the corresponding sequence of the SARS-CoV-S/RBT is sufficient to allow the activation of the human ACE2 receptor [28].

Coronaviruses can enter target cells effortlessly due to their ability to exploit many cell surface molecules such as proteins and carbohydrates. Lectins play a fundamental role in this process. For example, host calcium-dependent (type C) lectins have been recognized to play a central role in SARS-CoV-2 infection. Evidence suggests a specific intercellular role exerted by non-integrin 3-grabbing adhesion molecule (DC-SIGN) of dendritic cells. This is a type C lectin expressed on macrophages and dendritic cells that functions to recognize the high-mannose glycosylation patterns commonly found on viral and bacterial pathogens. Coronavirus protein S is highly glycosylated, thus, providing the virus with the opportunity to interact with host lectins such as Dendritic Cell (DC)/Liver/lymph node-specific intercellular adhesion molecule-3-grabbing integrin (L-SIGN). L-SIGN, which is expressed on liver and lung endothelial cells and has been reported as an alternative receptor for SARS-CoV and bat coronavirus type HCoV-229E [29,30].

The first demonstration of the possibility that SARS-CoV-2 interacts with the human ACE2 receptor is reported in the landmark study from the University of North Carolina at Chapel Hill [31,32]. The authors reported the substantially high risk of SARS-like bat coronavirus disease named SHC014-CoV circulating in Chinese horseshoe bat populations. This type of coronavirus has a high binding affinity with the ACE2 receptor [33,34]

The new SARS-CoV-2 virus expressed the bat coronavirus SHC014 spike in mouse-adapted SARS-CoV backbones.

Menachery et al. created a chimeric virus starting with the RsSHC014-CoV sequence that was isolated from Chinese horseshoe bats [34]. The chimeric virus encoded a different, zoonotic CoV spike protein in the context of the SARS-CoV mouse-adapted backbone. This new SARS-CoV-2 virus expressed the bat coronavirus SHC014 spike. Through the hybrid virus, the authors were able to evaluate the ability of the new spike protein to cause disease independently of other necessary adaptive mutations in its natural backbone [32].

The evidence showed at least two very interesting findings. The first was that group 2b viruses encoding the SHC014 peak in a wild-type backbone could efficiently use more orthologs of the human angiotensin II converting enzyme (ACE2) receptor than the unmodified SARS virus. The second was that group 2b viruses could replicate efficiently in primary human airway cells and that it was also possible to obtain in vitro viral titers equivalent to the epidemic strains of SARS-CoV. Once these results were translated in vivo, replication of the chimeric virus in the mouse lung demonstrated considerable pathogenesis. This led to the trials of immunotherapeutic and prophylactic modalities to cope with the SARS-CoV infection which had poor outcomes. In fact, both monoclonal antibodies and the vaccine approach failed to neutralize and protect against CoV infection using the new SARS-CoVS. Based on these results, the authors synthetically re-derived an infectious full-length recombinant SHC014 virus and demonstrated robust viral replication both in vitro and in vivo settings. This landmark report suggested 6 years ago that there was a potential risk of SARS-CoV re-emergence from viruses circulating in bat populations.

Recently the same group coordinated by Ralph Baric [35] studied the critical determinants of the ACE2 receptor that support SARS-CoV-2-ACE2 interactions during infection and replication of the preemergent 2B coronavirus (WIV). The Authors identified the key changes that lead to infection by creating a humanized murine ACE2 receptor (hmACE2) and provided evidence for the potential pan-virus capabilities of this chimeric receptor.

SARS-CoV-2 cannot infect mice due to incompatibility between its receptor-binding domain (RBD) and the murine ACE2 (mACE2) receptor. Since the mouse models of human ACE2 (hACE2) and viruses adapted to mice have shown limitations, the researchers developed another model that would allow evaluation of the pathogenetic phenomena that occur in human SARS-CoV-2 infection. For example, hACE2 transgenic mice are susceptible to unadapted SARS-CoV-2 viruses, but the pathogenesis observed in these mice showed that virus-induced encephalitis and multi-organ infection were not comparable to that observed in humans. Thus, Adams et al., to map the SARS-CoV-2 RBD and mACE2 interaction network, created a panel of mACE2 receptors, which have increasing levels of humanizing mutations. The study used predictive structural models that allowed identification of the minimal changes needed to restore replication [35].

The ACE2 receptor has structurally critical sites whose integrity determines its activity. The investigators worked at the level of three hot spots that determine ACE2 interaction: position K353 interconnects with SARS-CoV-2 binding residues G496, N501, and Y505, position K31 which forms a salt bridge with ACE2 residue K353 and links with SARS-CoV-2 Q493 and Y489, and position M82 which interconnects with RBD residues F486, N487, and Y489. These aforementioned interface hotspots are the critical molecular sites for the interaction between SARS-CoV2 and the receptor leading to virus entry. The authors demonstrated that divergent residue modifications in these hot spots significantly reduce the binding between humanized murine ACE2 (hmACE2) [33] and SARS-CoV-2 RBD. They recorded that five amino acid changes (N30D, N31K, S82M, F83Y, and H353K) in the SARS-CoV-2 RBD-ACE2 interaction hot spots lead to the modulation of infection and can re-establish infection in the hmACE2 models [35].

This study is crucial for the following reasons. The first is related to the fact that mouse models are essential for understanding the pathogenesis of coronaviruses and are a key resource for the preclinical development of vaccines and antiviral therapies. The second is that a detailed analysis of this study will allow the development of model systems to screen for emerging coronaviruses and to develop new treatments to combat infections [35].

### 1.5. The Role of ACE2 in COVID-19 Pathogenesis

The ACE2 receptor has been implicated in the pathogenesis of COVID-19, especially with regards to its potential effects on the most vulnerable patients presenting with cardiovascular co-morbidities. COVID-19 does not have the same impact on all members of the population. An exponential increase in the severity of the disease as well as mortality, due to devastating thromboembolic complications, occurs in patients over the sixth decade of life with comorbidities such as cardiovascular disease and diabetes.

The angiotensin-converting-enzyme 2 receptors (ACE2) serve as the attachment site of the SARS-CoV-2 spike protein to enter the lung epithelial cells [36]. Upregulation with increased ACE2 expression has been demonstrated in patients with cardiovascular disease and diabetes treated with angiotensin-converting enzyme (ACEI) inhibitors and angiotensin receptor blockers (ARBs). However, whether treatment with these agents can lead to greater COVID-19 severity has not been fully clarified.

Discussions related to the use of ACEI/ARBs have surfaced regarding the need to continue therapy in patients taking these drugs. The current recommendations are to discontinue the administration of these drugs, despite diverging opinions, which were not universally endorsed by experts due to the lack of strong evidence [37]. ACE2 not only plays a role in the pathogenesis of COVID-19 but also as a component of renin–angiotensin system signaling (RAS) localized throughout the body. Although the evidence has conclusively revealed that ACE2 receptors allow SARS-CoV-2 to enter cells, ACE2 plays a central anti-inflammatory role in RAS signaling by converting angiotensin II, responsible for the inflammatory process, into angiotensin 1–7, which leads to its anti-inflammatory effects [38]. A study performed on rodent lungs [39] showed that the reduced expression of ACE2 leads to a sequence of major proinflammatory processes, that are exacerbated by age, and result in dysregulation of RAS signaling throughout the body [40]. It is important to note that this typical inflammatory profile, even in accentuated forms, supports pathophysiological processes that represent the main feature of hypertension and diabetes, as well as being very widespread in old age [36]. The upregulation of the ACE2 receptor in subjects with diabetes and hypertension treated with ACI/ARBs must be seen as a restorative substrate that has a physiological function. The process that unfolds during SARS-CoV2 infection sees ACE2 receptors as a gateway for the virus to enter cells, while the reduction of ACE2 protective features in older people and those with CVD can potentially predispose them to more severe forms of the disease. The ACE2 receptor facilitates SARS-CoV2 infection while the fundamental anti-inflammatory function, linked to RAS signaling, is reduced because it is compromised in patients who develop COVID-19. In fact, data provided by the first SARS epidemic in 2003 demonstrated the double role of the ACE2 receptor, thus delineating the factors predisposing to the occurrence of the disease and its severity [41,42].

In SARS-CoV2 infection it is plausible that the higher expression of ACE2 leads to a greater predisposition to experience the disease. Epidemiological data from the South Korean population, where genetic testing has been widely used in individuals, reported higher numbers of infected among young adults [41] and those with increased ACE2 levels. In this regard, an Italian study, examining the severity of COVID-19 disease in the elderly population with CVD, hypothesized that a reduction in ACE2 levels due to aging and CVD coupled to the upregulation of the proinflammatory angiotensin II pathway are factors that likely predispose older individuals to severe forms of COVID-19. Therefore, younger people are more susceptible to viral infection, but older people are more likely to have severe disease manifestations [42].

SARS-CoV2 uses the ACE2 receptor in carrying out its infectious manifestation, thereby leading to a reduced expression of ACE2 on the cell surface and an upregulation of angiotensin II signaling in the lungs which results in the development of acute damage [38]. The consequence of these morphofunctional and biochemical changes can predispose elderly individuals with CVD, who have reduced levels of ACE2 compared to young people, to exaggerated inflammation and further reduction of ACE2 expression in the context of COVID-19. In these cases, the disease manifests itself with greater severity [43]. Observations suggest that older individuals, especially those with hypertension and diabetes, have reduced ACE2 expression and upregulation of proinflammatory angiotensin II signaling. Therefore, the morphofunctional and biochemical changes can be corrected by the increase in ACE2 levels induced by ACEI/ARB treatment [43].

**Figure 1:**
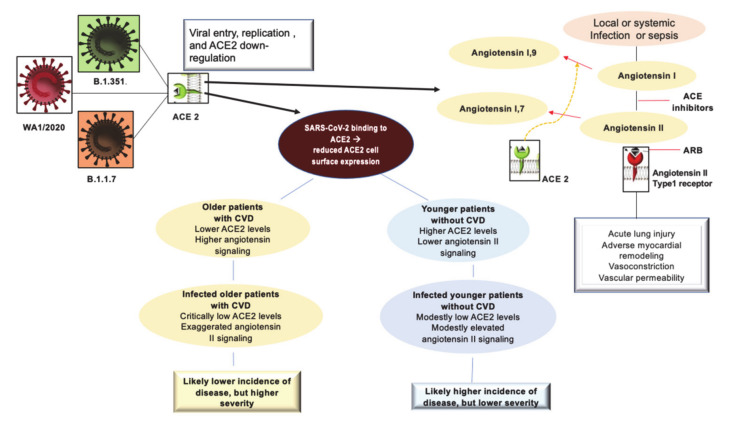
Depicts the interaction of SARS-CoV2 with the ACE2 receptor and the inflammatory profile pattern before and after Coronavirus 2019 (COVID-19) infection in patients with or without CVD. The initial entry of severe acute respiratory syndrome coronavirus 2 (SARS-CoV-2) into cells is shown with involvement mainly of type II pneumocytes. SARS-CoV-2 binds to its functional receptor, the angiotensin-converting enzyme 2 (ACE2). After endocytosis of the viral complex, surface ACE2 is further down-regulated, resulting in unobstructed accumulation of angiotensin II. Local activation of the renin–angiotensin–aldosterone system may mediate lung injury responses to viral insults. The elderly and the young may present with different pathophysiological profiles. The simplified scheme of the pre-infection inflammatory profile among predisposed older individuals compared to their younger counterparts is illustrated. Abbreviations: ACE2, angiotensin-converting enzyme 2; ARB, angiotensin-receptor blocker; CVD, cardiovascular disease; SARS-CoV-2, severe acute respiratory syndrome coronavirus 2.

First, it is possible to hypothesize that in COVID-19 disease, the binding of SARS-CoV-2 to ACE2 receptors acutely exacerbates this proinflammatory background, predisposing these subpopulations to greater severity and mortality of COVID-19 disease. Second, considering this hypothesis credible, a protective role of the antagonistic action of angiotensin II against acute lung injury associated with sepsis could be effective. This supports the use of continuous therapy with ACEI/ARB [1,44,45] (Figure 1).

Third, the aforementioned biomechanical modifications of the receptor, plausible with aging, should be investigated. Therefore, experiments on the functioning, the regulatory mechanisms of RAS, and the biomechanics of the receptors involved in these functions should be implemented. Specifically, the biophysical mechanisms underlying the associated remodeling of the lipid membrane remain to be clarified. They may be useful in the prevention of fatal lung complications caused by genetic variants of the Wuhan virus [26,27,28,32,34,35,46].

### 1.6. ACE Inhibitors and Angiotensin Type II Blockers Role in COVID-19 Severity

Tetlow et al. [47] did not identify any associations between ACE-I/ARB use and AKI, macrovascular thrombi, or mortality. Other studies [48,49] also supported the continuation of these drugs during hospitalization from COVID-19. Among those hospitalized, a large percentage are likely being administered either ACE inhibitors or Angiotensin II blockers, since epidemiological data reveal that cardiomyopathies, diabetes mellitus, and hypertension are the most frequent comorbidities found among those patients [50].

Although the upregulation of ACE2 expression, which can be altered by drug administration, has not been defined, it has nevertheless been associated with disease severity. Several preceding studies have demonstrated that the risk of developing COVID-19 after the administration of ACEi and ARBs increased significantly. This could be an indirect effect of overproduction of the circulating ACE2 transcripts in the cells [51,52]. As an example, Enalapril, which is a frequently used ACEi, was reported to increase ACE2 expression in the kidney [53].

Concerning possible therapeutical targets, ACE2 blockers have been developed, such as the small synthetic inhibitor N-(2-aminoethyl)-1aziridine-ethanamine (NAAE) [54]. It is able to bind ACE2 in its closed conformation so that molecular interaction between the viral particle and the receptor cannot be possible and fusion does not happen. Thus, NAAE could exert dual inhibitory effects: one on ACE2 catalytic activity and another on SARS binding [55]. Despite this, current research drives opinions towards a cautionary use of this agent.

## 2. Pathophysiology of Arterial and Venous Thrombosis

To date, the complete pathophysiology profile of arterial and venous thrombosis during COVID-19 disease has not yet been fully clarified. The literature reports prothrombotic abnormalities in patients with COVID-19. In a Chinese study [56] performed in the first phase of the SARS-CoV2 epidemic, 19 patients with COVID-19, who presented with critical clinical conditions, had elevated levels of markers of hypercoagulability such as D-dimer found in 100%, fibrinogen in 74%, and factor VIII in 100%. The dysregulation of the coagulation process included the presence of antiphospholipid antibodies in 53% of the population studied. Reduced levels of protein C, protein S, and antithrombin were noted in all patients. Complications such as stroke, arterial ischaemia, and VTE accompanied the coagulation disorder.

Zaid et al. studied 115 patients with COVID-19 disease reporting that SARS-CoV2 directly interferes with platelets. Viral RNA and high platelet-associated cytokine levels were found in the platelets of all study participants. These abnormalities were not related to the severity of the disease because in 71 infected individuals the disease manifested in a non-serious manner while for 44 patients’ hospitalization was required for critical clinical conditions. Specific tests performed on platelets showed aggregation at lower than expected thrombin concentrations [57].

Nicolai et al. examined the autopsy findings of 38 individuals who died with COVID-19 which showed that histopathological changes in coagulation were marked in the vessel microcirculation. The abnormalities recorded were microvascular thrombotic formations, and neutrophil extracellular traps characterized by networks of extracellular neutrophil-derived DNA and polymorphonuclear neutrophil (PMN)-platelet aggregates [58]. The authors compared the peripheral blood of patients with COVID-19 with that of healthy patients. In vitro responses on peripheral blood samples from the three infected patients exhibited excessive platelet and neutrophil activation, as assessed by degranulation and integrin IIb-IIIa activation and immunofluorescence, compared to healthy control patients’ samples.

### 2.1. The Inflammatory Response during SARS-CoV2 Infection and Thrombotic Complication

Histopathology of SARS infection Cov2 is distinguished from that caused by other viruses with tropism for the respiratory tract. SARS-CoV2 leads to direct damage of endothelial cells characterized by dense perivascular infiltration of T lymphocytes combined with aberrant activation of macrophages. The excessive and uncontrolled inflammatory response, endothelial cell apoptosis, thrombotic microangiopathy, and angiogenesis are other distinctive histopathological features that denote the aggressiveness of SARS-CoV2, which may be responsible for clinically severe forms of COVID-19 thus conferring disease characteristics not comparable to any other viral respiratory disorder [59]. One significant finding that emerges in the evaluation of the pathophysiology of thromboembolism in COVID-19 versus non-COVID-19 disorders is the possibility that the coagulation alterations are mediated more by platelet-dependent activation and intrinsically related to viral-mediated endothelial inflammation. As noted a distinguishing feature of thrombosis during SARS-CoV2 infection is the exacerbated hypercoagulability associated with increased concentrations of coagulation factors, acquired antiphospholipid antibodies, and reduced concentrations of endogenous anticoagulant proteins [56].

Patients with COVID-19 who develop more severe systemic inflammation and more critical respiratory dysfunction have a higher prevalence of thrombotic complications. Lodigiani et al. reported 388 patients hospitalized with COVID-19 including 16% with serious clinical conditions. Despite the use of low molecular weight heparin (LMWH) for thromboprophylaxis in all patients in the ICU and 75% of those not in the ICU, symptomatic VTE occurred in 4.4% of patients, ischemic stroke in 2.5%, and MI in 1.1% [60].

Given the knowledge we have, there is still no clarity on the extent to which SARS-CoV-2 increases the risk of thromboembolism. A study performed in the United Kingdom compared 1877 patients discharged from hospital after COVID-19 disease and 18,159 hospitalized for a non-COVID-19 disease reported no difference in hospital-associated VTE rates (4.8/1000 vs. 3.1/1000; odds ratio, 1.6 [95% CI, 0.77–3.1]; *p* = 0.20) [61]. One point to clarify is whether the high rate of VTE is specific to patients who develop COVID-19 or if VTE is mainly occurring in patients as a complication associated with severe critical disease [61]. These results are in line with a recent meta-analysis that included 41,768 patients in whom VTE was assessed in COVID-19 versus non-COVID-19 cohorts. The authors did not record a significant statistical difference for overall risk of VTE (RR 1.18; 95%CI 0.79–1.77; *p* = 0.42; I^2^ = 54%), pulmonary embolism (RR 1.25; 95%CI 0.77–2.03; *p* = 0.36; I^2^ = 52%) and deep venous thrombosis (RR 0.92; 95%CI 0.52–1.65; *p* = 0.78; I^2^ = 0%). A difference was reported after analyzing the subgroups of patients who were admitted to the intensive care unit (ICU). Critically ill patients had an increased risk of VTE in the COVID-19 cohort compared to non-COVID-19 patients admitted to the ICU (RR 3.10; 95% CI 1.54–6, 23), which was not observed in cohorts of non-ICU patients (RR 0.95; 95% CI 0.81–1.11) (P interaction = 0.001) [62].

### 2.2. Management of Thrombosis in COVID-19 Patients

**Figure 2:**
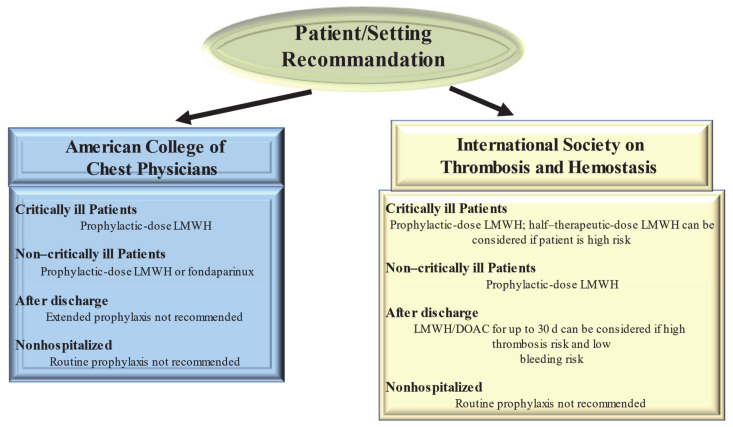
Current Guideline Recommendations for Venous Thromboembolism Prevention in patients With Coronavirus Disease 2019. Abbreviations: DOAC, direct oral anticoagulant; LMWH, low-molecular-weight heparin.

There are no international guidelines that direct the prevention and treatment of thrombotic complications in COVID-19 patients. Both published and ongoing studies testing interventions to prevent thrombosis complications in COVID-19 are based on the evidence reported in current clinical guidelines about VTE prophylaxis in acute COVID-19 infections. Therefore, pending the results to be provided by the completion of ongoing trials, guidelines for the treatment of thrombotic complications in patients with COVID-19 disease are derived from medical recommendations in the coagulation disorder populations (Figure 2). However, the crucial point that remains to be clarified is whether these guidelines are also optimal for the treatment of thrombosis due to COVID-19 [63,64,65].

Guidelines from the American College of Chest Physicians (ACCP) suggest (in the absence of contraindications) prophylaxis with LMWH or fondaparinux rather than unfractionated heparin or direct oral anticoagulants (DOACs) for all hospitalized COVID-19 patients [63] Clearly the optimum choice of the drug to be taken is constrained by the incomplete knowledge of the possible interference of CoV 2 SARS with the medicament. So, the 40 mg dose of LMWH for injection once a day and the 2.5 mg dose of fondaparinux are preferred over the administration of unfractionated heparin injected subcutaneously 2–3 times a day thus limiting the caregiver’s contact with infected patients. In addition, these drugs are preferred over DOACs because of drug–drug interactions with antiviral agents. Both are substrates of the P-glycoprotein and/or cytochrome P450-based metabolic pathways. Thus, concomitant administration of DOACs and antiviral drugs has the potential to sharply increase DOAC anticoagulant plasma levels, thus increasing hemorrhagic risk.

Given the high incidence of VTE, the proposed therapeutic dose to be used for standard thromboprophylaxis in critically ill patients with COVID-19 was either double or single-dose administration of LMWH. The ACCP guidelines suggest the standard dose LWMH in the absence of new clinical trial data [64]. Guideline-Directed Medical Therapy (GDMT), which was established by the International Society on Thrombosis and Hemostasis (ISTH), suggested that half-therapeutic-dose LMWH (1 mg/kg daily) can be considered for prophylaxis in high-risk patients with COVID-19. A 50% higher dose can be considered in patients with severe obesity (BMI ≥ 40 kg/m^2^). However, it remains to be clarified which is the best dosage for optimal prophylactic therapy [65]. The results of ongoing randomized controlled trials (REMAP-CAP, ACTIV-4, and ATTACC), comparing therapeutic-intensity anticoagulation with prophylactic-intensity anticoagulation for patients with COVID-19-related critical illness, are awaited to establish optimal antithrombotic prophylactic therapy [66]. Considering the pathophysiology of thromboembolism in COVID-19 is characterized by platelet hyperreactivity, another point under discussion with RCTs initiated is the evaluation of administering an antiplatelet agent for therapeutic prophylaxis.

High-risk patients hospitalized for COVID-19 have a high possibility of developing VTE that persists after discharge [65]. However, for the latter, no specific recommendations for post-discharge thromboprophylaxis have been established by the ACCP [64]. In contrast, the ISTH recommendations for post-discharge thromboprophylaxis suggest the use of LMWH or a DOAC for all high-risk hospitalized patients with COVID-19 who have a low risk of bleeding. Patients with COVID-19 considered to be at high risk include those with age ≥ 65 years, presence of critical illness, cancer, previous VTE, thrombophilia, severe immobility, and elevated D-dimer (>2 times the upper limit of normal). ISTH recommendations suggest a duration of 14 to 30 days for post-discharge thromboprophylaxis, although the ideal administration period remains to be clarified [65].

For patients with COVID-19 disease, no diagnostic protocols have been established for thromboembolic complications, such as pulmonary embolism and MI, so the methods to be used should be those validated for patients without COVID-19. Given the lack of evidence to support the benefit, routine ultrasound checks for VTE surveillance are not recommended. For patients with COVID-19 diagnosed with arterial or venous thrombosis, we recommend treatment according to current established guidelines. These recognize the benefits of LMWH administration in hospitalized patients. In the outpatient setting, DOAC administration is recommended [58]. There are currently no recommendations issued by ISTH and ACCP to support measuring the D-dimer to screen for VTE or to establish the intensity of prophylaxis or treatment [64,65].

## 3. COVID-19 Vaccines Administration vs. Thrombosis and Variant. The New Challenge

### 3.1. Nucleoside-Modified RNA Encoding the SARS-CoV-2 Spike

Several studies have demonstrated the substantial role of the SARS-CoV-2 spike protein that binds to ACE2 receptors on target cells during viral entry. Studies performed on convalescent patients have highlighted the central role that the spike protein plays as an immunodominant antigen triggering the host response, mediated by both antibody and T lymphocytes [67].

Concerns about the rapid spread of the COVID-19 pandemic have favored the registration of numerous randomized clinical trials (RCTs) using different vaccination platforms in order to evaluate their efficacy and safety. Evidence has shown that the use of rapid response genetic platforms mRNA [67,68], adenoviral vector vaccines [69,70], inactivated viruses [71,72], and adjuvanted spike glycoprotein [73] resulted in neutralizing antibody responses after immunization.

The particular biological characteristics of mRNA synthesized in vitro may explain the superior efficacy of an ideal non-viral gene replacement tool leading to many intrinsic benefits [67,68]. First, quick protein assembly and well-regulated primary cell transduction. Second, mRNA-based therapy avoids harmful side effects, such as its incorporation into the cellular genetic substrate, which can ultimately limit the clinical application of most virus- and DNA-based vectors [67,68].

Although the use of in vivo gene transfer therapy was first applied almost twenty years ago [74,75], its usage as a vector for introducing genetic material into animals or even into cultured cells has been very limited. Indeed, the reports of Gilboa [76] and Pascolo [77] focused on the use of mRNA for vaccination purposes mainly directed at the development of cellular and humoral immune responses through antigen-encoding transcripts that were administered in vivo or delivered to dendritic cells (DC) ex vivo.

Several studies conducted at the beginning of the year 2000 [78,79,80,81,82] have shown that RNA interferes with the cell-mediated adaptive immune response (pathogen and antigen-specific response) by activating the cells of the innate immune system (non-specific response). In particular, the action of the mRNA is directed towards the Toll-like receptors (TLR) and specifically for the cellular subgroups TLR3, TLR7, and TLR8. It should be noted that compared to gene replacement, RNA showed greater immunogenicity and was associated with greater efficacy, highlighting a key role in the immune response.

Only 1 published study compared the in vitro immune response between nucleosides and modified nucleosides. The use of incorporated pseudouridine (Ψ), 5-methylcytidine (m5C), N6-methyladenosine (m6A), 5-methyluridine (m5U) or 2-thiouridine (s2U) in the transcript affected the immune response of most TLRs with a substantial loss of their activation [83]. Progressing to testing nucleoside-modified mRNAs to evaluate their translation potentials and immune characteristics in vivo, Hornung et al. [83] demonstrated that the 5′-triphosphate end of RNA produced by viral polymerases is accountable for retinoic acid-inducible protein I (RIG-I)-mediated detection of RNA molecules. Identification of 5′-triphosphate RNA is repealed by capping the 5′-triphosphate end or by nucleoside modification of RNA, including the use of s2U and Ψ.

The major implication of these findings led to in vitro transcripts containing nucleoside modifications not only translatable but also capable of activating an immune response in vivo. Therefore, it was possible to subsequently develop mRNA with the function of a dual therapeutic tool for both gene replacement and vaccination. Evidence reported by Kariko et al. [84,85] on the in vitro incorporation of pseudouridine, a modified nucleoside present in mRNA, has suggested that it improves RNA translation capacity but also suppresses RNA-mediated immune activation in vitro and in vivo.

### 3.2. RNA Vaccine Platform

In January 2020 the RNA sequence of the new coronavirus SARS-CoV2 was introduced in the RNA vaccine platform to allow rapid development of the vaccine in response to the worsening spread of the pandemic. The advantages of vaccines that use RNA are manifold and related to the incorporation of pseudouridine [84,85]. They offer both greater flexibility during vaccine antigen design and expression as well as the ability to imitate viral antigen structure and expression during native infection. One of the characteristics that make them innovative is the lack of integration of viral RNA, which is necessary for protein synthesis, in the cell’s genome. In fact, the viral genome is transiently expressed, then metabolized and eliminated by the natural mechanisms of the organism, giving these vaccines greater safety [68,69,70,71,72]. As a rule, vaccination with RNA can stimulate a vigorous innate immune response eliciting B and T cell-dependent activity. RNA leads to the expression of the vaccine antigen in host cells and, as demonstrated in specific mRNA vaccines, could address considerable medical demand in the area of influenza prophylaxis [86].

The immunogenic benefits associated with the in vivo administration of 1-methyl pseudouridine-containing mRNA including superior translational capacity and biological stability were established in a landmark paper from Kariko et al. [84,85]. The same research paper demonstrated lower serum levels of interferon-α (IFN-α) elicited by modified mRNA in mice models with respect to unmodified ones, thus potentially reducing exaggerated systemic inflammation and increasing safety. The improved efficacy of nucleoside-modified RNA (modRNA) encoding the SARS-CoV-2 full-length spike modified by two proline mutations is almost certainly due to its superior immune response [85]. Studies conducted in the United States and Germany have reported substantially higher elicited SARS-CoV-2 neutralizing antibody titers and robust antigen-specific CD8+ and Th1-type CD4+ T-cell responses against nucleoside modified mRNA delivered in lipid nanoparticles [86,87,88].

There are two widely administered mRNA vaccines, BNT162b2 (Pfizer–BioNTech) and mRNA-1273 (Moderna). Administration of a two-dose regimen of BNT162b2 conferred 95% protection against COVID-19 in phase 3 trial participants (n = 21,720), aged 16 years and older (95%; confidence interval, 90.3 to 97.6). The group of participants assigned to receive BNT162b2 recorded 8 cases of COVID-19 disease with onset at least 7 days after the second dose while in the group of individuals assigned to placebo there were 162 cases of COVID-19 disease. Evidence observed from the analysis of subgroups defined by age, sex, race, ethnicity, baseline body mass index, and the presence of coexisting conditions reported similar efficacy of the vaccine with percentages between 90 and 100%. It is important to note that among 10 cases of severe COVID-19 with onset after administration of the first dose, 9 were reported in recipients of the placebo dose and 1 recipient with the BNT162b2 dose. The safety profile of BNT162b2 was very high as evidenced by the short-term appearance of mild-to-moderate pain at the injection site, fatigue, and headache. The side effects were low and comparable to those recorded in the placebo dose recipients. Furthermore, they were equivalent for a median of 2 months when comparing BNT162b2 with that of other viral vaccines [89].

Similar results were reported in phase 3 of the randomized, observer-blind, placebo-controlled trial after administration of mRNA-1273 (100 μg in 15,210 participants) [90]. The efficacy of the mRNA-1273 vaccine was recorded at 94.1% (95% CI, 89.3 to 96.8%; *p* < 0.001) in the prevention of COVID-19 disease, including severe disease. A double dose of mRNA-1273 was administered to more than 96% of vaccine recipients and only 2.2% had serological, virological, or both evidence of SARS-CoV-2 infection. Symptomatic COVID-19 disease was confirmed in 185 recipients of the placebo dose versus 11 recipients of mRNA-1273 dose.

Recipients of mRNA-1273 reported only transient local and systemic side effects and no safety concerns were recorded. A critical illness from COVID-19 occurred in the 30 recipients of the placebo dose (with one death) and in no participant who was administered the mRNA-1273. It is important to clarify that current data on the messenger, derived from laboratory studies, have demonstrated the efficacy of RNA (mRNA) vaccines against SARS-CoV-2 variants. Researchers exposed serum samples from immunized individuals to genetically modified versions of related variants and then measured neutralizing antibody titers [90].

### 3.3. SARS-CoV-2 and Vaccine

We searched PubMed for research articles published by the launch of the database until April 30, 2021, indicating no language restrictions and using the terms “SARS-CoV-2”, “vaccine”, and “clinical trial”. We identified published clinical trial data on seven SARS-CoV-2 studies and vaccines.

Four recombinant viral vectored vaccines have been tested in phase I/II clinical trials [91,92,93,94,95,96,97,98]. Phase I and II trials were represented in the same study by two parts with different patients subsets. The vaccine ChAdOx1 nCoV-19 (AZD1222), known as AstraZeneca vaccine, was developed by the Oxford University and is constituted with an adenoviral vector inactivated (unable to replicate) chimpanzee ChAdOx1 replication, containing the antigen glycoprotein gene structural surface SARS-CoV-2 (protein spike; nCoV-19). This vaccine is one of the more extensively studied following the first UK Phase 1 clinical trial published on 23 April 2020 [92]. To date three more randomized controlled trials of the candidate vaccine have been initiated in the UK (COV002), Brazil (COV003), and South Africa (COV005). Recently a further phase 1/2 study was carried out in Kenya.

A pooled interim analysis of four trials (COV004) showed the safety and efficacy of the ChAdOx1 nCoV-19 vaccine (Oxford-AstraZeneca COVID-19 vaccine), (Covishield or Vaxzevria). In recipients of two standard doses of Vaxzevria, the vaccine efficacy was 62.1% and in recipients given a low dose followed by a standard dose, the efficacy was 90.0%. The overall efficacy of the vaccine after administration of both doses in the population studied was 70.4%. There were ten cases of COVID-19 that required hospitalization 21 days after the first dose, all in the control population. Two patients were in serious condition and one died. The authors recorded 175 serious adverse events that occurred in 168 participants, of which 84 events occurred in recipients of the Oxford-AstraZeneca COVID-19 vaccine and 91 in the control group. Concern relating to clot formation or the occurrence of bleeding episodes were not suggested across the analysis of these 4 RCTs [91].

The immune response after vaccine administration in participants who received two doses of the vaccine was very effective. In particular, the specific objectives of phase 3 RCT were the evaluation of humoral and cellular safety and immunogenicity concerning both a single dose and two-dose regimen in adults over 55 years of age. Median peak anti- SARS-CoV-2 IgG responses 28 days after the boost dose were similar across the three cohorts (including two groups of patients aged 18–55 and one group enrolling >55 years old patients). Furthermore, neutralizing antibody titers after a boost dose were similar across age groups. Within 14 days of boost dose administration, a total of 208 of 209 (>99%) recipients of the booster dose of ChAdOx1 nCoV-19 had neutralizing antibody responses. T cell responses peaked on day 14 following a standard single dose of ChAdOx1 nCoV-19 [90,91].

The Ad26.COV2. S vaccine, known as Johnson&Johnson/Janssen COVID-19 vaccine, is composed of a recombinant, replication-incompetent human adenovirus type 26 (Ad26) vector that encodes a full-length, membrane-bound SARS-CoV-2 spike protein in a prefusion-stabilized conformation [95,96,97]. At least 14 days after single-dose administration, Ad26.COV2. S conferred protection against both symptomatic COVID-19 infection and asymptomatic SARS-CoV-2 infection. The level of efficacy remained stable at 28 days after administration with an efficacy of 66.1% (adjusted 95% CI, 55.0 to 74.8). COVID-19 disease occurred in 66 recipients of the administration dose of Ad26.COV2. S compared to 193 for the placebo dose [98]. The results of the administration of Ad26.COV2. S vaccine has demonstrated efficacy against COVID-19 clinical disease with severe-critical manifestation, including hospitalization and death. Evidence suggested a high level of efficacy after administration of Ad26.COV2, which was greater against severe-critical COVID-19 disease and reached a rate of 76.7% for onset at ≥14 days [adjusted 95% CI, from 54.6 at 89.1]. At 28 days in participants receiving the single dose the reported efficacy was 85.4% [adjusted 95% CI, 54.2 to 96.9] for onset at ≥28 days).

However, the unexpected data were related to the immunogenic response to Ad26.COV2 vaccine against the South African variant 20H/501Y.V2. Out of 91 cases of patients in which the virus variant was sequenced and confirmed, 86 (94.5%), showed vaccine efficacy against moderate to severe-critical COVID-19 that reached 52.0% and 64.0% with onset of at least 14 days and at least 28 days after dosing, respectively. The efficacy against severe-critical COVID-19 disease reached 73.1% and 81.7% at 14 days and 28 days respectively after the single dose of Ad26.COV2. Evidence supported a level of safety comparable to that of other COVID-19 vaccines that progressed to phase 3 studies. The reactogenicity was greater with the administration of Ad26.COV2. S compared to the placebo dosage; however, it was generally mild to moderate and transient. Note that the incidence of severe adverse events was similar between the two populations of participants (vaccine and placebo group) with three deaths occurring in the vaccine group (but none of them was related to COVID-19 infection). No episodes attributable to thrombotic or haemorrhagic phenomena were reported [98].

Vector-based adenovirus (Ad) 5 (CanSino Biological/Beijing Institute of Biotechnology, China) [69,93] was administered in a single dose and resulted in the production of neutralizing antibodies which increased significantly on day 14 and peaked 28 days after vaccination. A specific T-cell response in a dose-dependent manner peaked at day 14 after vaccination. However, of note, the vaccine demonstrated lower immunogenicity in participants over the age of 55. Administration of adenovirus type-5 vectored COVID-19 recorded no serious adverse event within 28 days post-vaccination. An equal rate of side effects was reported in the three groups studied. Reactogenicity was evident in the first 7 days after administration in 30 (83%) recipients of a low dose vaccine, in 30 (83%) recipients of a medium dose, and in 27 (75%) recipients of a high dose, respectively [93].

A heterologous recombinant adenovirus (rAd26 and rAd5)-based vaccine, Gam-COVID-Vac (Sputnik V) [70,94] showed efficacy and safety from the interim analysis of a phase 3 RCT. Gam-COVID-Vac is a combined vector vaccine because it consists of rAd type 26 (rAd26) and rAd type 5 (rAd5). Both adenoviruses carry the gene for the SARS-CoV-2 full-length glycoprotein S (rAd26-S and rAd5-S). The administration of rAd26-S and rAd5-S is carried out (intramuscularly) separately with an interval of 21 days. The results of the Phase 1/2 clinical trials showed that the Gam-COVID-Vac vaccine was well tolerated and highly immunogenic in healthy participants. Vaccine efficacy of Gam-COVID-Vac reached 91.6% (95% CI 85.6–95.2) with few tolerable side effects (7485 [94.0%] of 7966 total events). Although 45 (0.3%) of recipients of this vaccine (n = 16,427) and 23 (0.4%) of recipients of the placebo dose recorded serious adverse events; however, none were deemed associated with vaccination. There were four deaths during the study. Three (<0.1% of a total of 16,427) were participants from the vaccinated population and one (<0.1% of the total of 5435) received a placebo. None of these deaths were considered related to the vaccine.

Chinese researchers worked on two inactivated viral vaccines [71,72]. Two SinoPharm vaccines demonstrated the neutralization of antibody responses in participants, aged 18–59, who received the first SinoPharm vaccine (Wuhan Institute Biological Products Co Ltd /SinoPharm, Wuhan, China). The immune response was dose-dependent. The second SinoPharm product (Beijing Institute of Biological Products-Sinopharm-China National Biotec Group Co, Beijing, China) elicited neutralizing antibody response in adults aged 18–59 and 60 years and older. However, the latter vaccine showed lower neutralizing antibody titers in older adults after two doses.

This phenomenon is related to the presence of antibodies before vaccination which was present, albeit with variable vaccine titrations, in the three study groups. Only 25% of participants in the low dose group, 37% of participants in the medium-dose group, and 63% of the high dose recipients, who had pre-existing high immunity to Ad5, had at least a fourfold increase in neutralizing antibody titer on day 28 after vaccination. Multivariable analysis showed that the pre-existing high Ad5 neutralizing antibody titers impaired post-vaccination neutralizing antibody seroconversion and highlighted a different immune response in relation to the age of the recipients. The impairment of serum conversion was independent of the dose of vaccine administered in the three groups (the low-dose, the medium-dose, and the high-dose ones). However, recipients aged 45–60-year-old appeared to have lower neutralizing antibody seroconversion than younger recipients. In the latter, Ad5 neutralizing antibodies were significantly enhanced after vaccination [71,72]

Finally, a clinical study of a vaccine NVX-CoV2373 (Novavax Inc. (NVAX), Gaithersburg, MD, USA) assembling nanoparticles consisting of adjuvant trimeric spike glycoproteins from severe acute respiratory syndrome coronavirus 2 (SARS-CoV-2) recorded preliminary results. The vaccination schedule included the administration of two doses 3 weeks apart in healthy adults less than 60 years of age. Evidence suggested good tolerance for this vaccine which induced neutralization responses greater than those measured in serum samples from convalescent symptomatic susceptible patients [64].

### 3.4. Vaccines and Immunogenicity Against Genetic Variants

Given the worldwide spread of the genetic variants of SARS-CoV-2 and the increasing number of cases of COVID-19 disease, the recurring question today is whether the vaccines currently administered will be effective against the mutated viral variants. Since in the case of vaccines efficacy is based on immune responses, it is evident that the patient may have a reduced immune response to viral variants.

Concerns related to less immunogenicity of vaccines emerged at the end of January 2021, simultaneously with the effects of SARS-CoV-2 mutagenic potential including the strong spread of South African variant B.1.351 [99].

Despite the many mistakes that viruses can make by replicating, we are not aware of any vaccines against viral diseases, other than seasonal influenza, which have required regular updates on the basic constitution due to changes in the viral genome. For example, despite the frequent mutations recorded by the hepatitis B virus, the vaccine continues to guarantee safety and efficacy in the vaccinated population.

The progression of vaccination is more rapid in high-income countries. Unfortunately, the majority of the world population lives in low-middle-income countries where the mass vaccination programs remain restricted or exclusive. As persistent infections and viral replication create the possibility of high-frequency mutations of the SARS-CoV-2 genome, we must seek to homogeneously extend vaccine administration without any economic or social class distinctions.

### 3.5. Current Knowledge

Although the term vaccine resistance has been used by experts in the field to describe the reduced efficacy of COVID-19 vaccines against some variants, this term can be inaccurate. In fact, the concept of drug resistance is more commonly aimed at antibiotics that are used to kill or are capable of inhibiting the growth or reproduction of bacteria. In the case of vaccines, the administration has not taken place, so the person cannot be resistant but can have a reduced immune response.

Vaccines administered against COVID-19 are engineered from the SARS-CoV-2 spike protein of the original virus called Wuhan-hu-1 [100] which is used by the virus to bind and infect host cells. Emerging data from COVID-19 disease suggest that variants of the “parent” virus appear to be more transmissible or more lethal than Wuhan-hu-1 and may contain mutations in the spike protein causing vaccine efficacy problems. All of the randomized controlled trials evaluating Pfizer-BioNTech and Moderna vaccines, the first to receive emergency use authorization (EUA), were conducted primarily in the United States. Authorization occurred before any cases of infection attributable to variant B.1.351 or others emerged, raising the possibility of reduced efficacy of Pfizer-BioNTech and Moderna in the USA [67,68,69,70,71,72,73,74,75,76,77,78,79,80,81,82,83,84,85,86,87,88,89,90].

Regarding viral vector vaccines or the vaccine that uses a nanoparticle, the evidence provided by the RCTs of Novavax [101], Janssen/Johnson&Johnson, and AstraZeneca in South Africa, where variant B.1.351 is widespread, have raised uncertainty about the effectiveness of vaccines. The concern is related to the fact that variant B.1.351 represents practically all the SARS-CoV-2 circulation in South Africa and a reduction in the efficacy of these vaccines has been recorded in comparison to other countries where the variant was not dominant [91,98].

Scientists working on the efficacy of mRNA vaccines have produced evidence about the SARS-CoV-2 variants that are derived from laboratory studies. Researchers tested the serum of people immunized to the modified protein spike variant of the virus and subsequently measured the antibody titers produced. Such studies have consistently reported that vaccines generated lower levels of neutralizing antibodies against the SARS-CoV-2 variants than the antibody titer that was produced against the older and more common variants.

In one study [102] serum samples, from individuals immunized with 2 doses of Pfizer-BioNTech vaccine were evaluated and tested against the recombinant virus which contained S-glycoprotein mutations similar to those found in variant B.1.351. The authors reported that compared to the neutralization of USA-WA1/2020, the neutralization of B.1.1.7-spike + E484K and B.1.526-spike viruses was approximately equivalent while the neutralization of B.1.429-spike was slightly lower. For the latter, a direct influence exerted by the L452R mutation has been hypothesized, which appears to be under positive selective pressure. The authors suggested that, compared to the previously reported neutralization of B.1.1.7-spike, the additional E484K mutation, which is also present in B.1.351 and B.1.526 lineages, caused few compromises to neutralization.

Another report [103] measured neutralization antibody activity using serum samples of recipients of the mRNA-1273 Moderna vaccine belonging to phase 1 of the trial. One week after the individuals received the second dose of mRNA-1273 Moderna vaccine, neutralizing antibody titers induced by a recombinant virus bearing the B.1.351 spike protein were 6-fold lower than those induced by a recombinant virus bearing the original Wuhan-Hu-1 spike protein. However, the elicited antibody response may still be sufficient to protect against COVID-19, or at least the more severe forms of COVID-19.

### 3.6. Immunogenicity and Variants. Where the Effectiveness of Vaccines Ends

To date, optimal antibody protection levels have not yet been determined for SARS-CoV-2 infection. There are favorable data in regard to the immune cell response mediated by virus-specific helper T cells and cytotoxic T cells induced by mRNA vaccines which in addition to neutralizing antibodies, counteracts the infection [104].

Today, we do not have immune correlates of protection against the variants of SARS-CoV2 and only the massive administration of vaccines in the population will be able to provide evidence if they are effective in preventing the contagion of the infection caused by the variants. As of today, we only know the efficacy of selected vaccines and for selected variants (Table 1).

Indeed, the opposite has happened. Individuals who had been vaccinated were hospitalized because they had COVID-19 infection caused by the mutated virus [106]. A frustrating situation is suggested by the discouraging results from the Phase 2 trials of the Oxford-AstraZeneca vaccine administered in South Africa. In the study participants, it was recorded that the vaccine did not prevent mild to moderate COVID-19 disease caused by variant B.1.351 [107]. Following the findings, the vaccination schedule based on the administration of AstraZeneca was modified [108]. Analyzing this report it emerges that it was not designed to determine if the vaccine leads to protection against severe forms of COVID-19 or not. A potential bias was the number of participants (n = 2000) who were young, mean age 31, healthy and at low risk of developing severe COVID-19 disease regardless of inclusion in the vaccine or non-vaccine group.

Studies by Novavax and Janssen have provided more evidence from the administration of their vaccines in South Africa than the Oxford vaccine/AstraZeneca. The results demonstrate lower efficacy rates for Novavax and Janssen in participants enrolled in randomized controlled trials in South Africa compared to studies in other countries. Nevertheless, the phase 3 results in recipients of the Janssen vaccine showed that the likelihood of being hospitalized for a severe form of COVID-19 was lower than those who received the placebo dose. The phase 3 results of the Novavax vaccine RCT, not yet published, could point in the same direction [73,109].

The reference point for an analysis of the real situation on the progression of COVID-19, both in reference to the spread of the infection/number of cases and the need for hospitalization or intensive care, is Israel [110] but the Kingdom of Bhutan a land locked country in the Eastern Himalayas has been a case study for low viral diffusion. As of 11 May 2021, the World Health Organization WHO reports 20 new cases per day, 1241 total cases (PCR-positive COVID-19 patients, both symptomatic and asymptomatic), and 1 death.

In Israel, the country which is the world leader in percentage of the population vaccinated, the number of COVID-19 patients began to decline in mid-January 2021. The effects of the mass vaccination program are evident in the drastic reduction in the need for hospitalization and a reduction in the absolute number of infections in older individuals who were the highest priority for vaccination [111]. An analysis of the hospitalization trend shows that in one week the percentage of patients requiring hospitalization for a severe form of COVID-19 decreased from 36% to 29%, compared to the previous 3 weeks. As variant B.1.1.7, first isolated in the UK, is now the dominant variant of SARS-CoV-2 in Israel as well as in the United Kingdom it is evident that this variant does not seem to influence the production of neutralizing antibodies after the administration of the Pfitzer Biointech vaccine to the same extent as it did for B.1.351 [111].

The same outcomes were recorded from a report performed in the United Kingdom in which researchers compared the Pfizer-BioNT and Oxford-AstraZeneca vaccines. They demonstrated that the latter achieved efficacy in preventing COVID-19 disease in 94% of recipients compared to 85% of Pfizer-BioNT vaccine recipients. Hospital admissions were therefore reduced in the 28–34 days after a single dose. The results of this study suggest a postponement of the administration of the second dose until 12 weeks after the first dose [112].

Generally, human vaccines have the characteristic of reducing the manifestations of the disease and in general also the transmission. This ability has not yet been fully demonstrated for vaccines against COVID-19. Therefore, another critical issue could involve asymptomatic vaccinated individuals infected by variants which represent a reservoir of contagion and diffusion of the variants. The increased risk of these individuals lies in the fact that the absence of symptoms does not prevent the spread of the infection because they are capable of infecting unvaccinated individuals.

Recent evidence suggests a reduction in transmission. An Israeli study conducted at the Hadassah Hebrew University Medical Center (HHUMC) evaluated viral transmission in healthcare professionals. Individuals were immunized with two doses, 21 days apart, of Pfizer-BioNTech vaccine and were subjected to regular testing with a PCR test and two rapid tests to identify the percentage of infected individuals in the populations of asymptomatic and symptomatic patients. The results revealed a 70% reduction in infection in the two populations after 21 days from the administration of the first dose of vaccine and an 85% reduction after administration of the second dose [113]. Vaccines efficacy was also demonstrated to be active during B.1.1.7 variant surge, constituting 80% of PCR-positive cases, as reported by the Ministry of Health of Israel in COVID-19 research reports [113]. These findings could be supported by a Pfizer-BioNTech-sponsored study not yet peer-reviewed in which the vaccine was 94% effective in reducing transmission of asymptomatic SARS-CoV-2 infection [114,115].

Uncomfortable data emerge after the identification of SARS-CoV-2 variant B.1.617.2 (Delta). This viral variant was identified in India in late 2020 and was subsequently detected in 60 other countries. The main feature of variant B.1.617.2 is the potentially higher transmission speed than other variants.

On 12–18 May 2021, the Oklahoma State Department of Health (OSDH) Acute Disease Service (ADS) registered the presence of the delta variant. A total of 21 SARS-CoV-2 delta specimens, temporally and geographically clustered in central Oklahoma, were sequenced by the OSDH Public Health Laboratory (PHL). The data that emerged from public health surveillance indicated that people infected with delta viral variants were associated with a local gymnastics facility [116].

The checks put into place by the OSDH ADS and by members of the staff of the local health department led to the identification of people who tested positive for the delta variant with the search for contacts. Forty-seven cases of COVID-19 emerged that developed in an age group between 5 and 58 years. Of these 21 delta variant cases belonged to the primary cluster and 26 other cases were epidemiologically linked and were associated with the primary outbreak. Distribution was essentially restricted to 10 of 16 gymnast cohorts* and three staff members. However, the spread of the delta variant was recorded in seven (33%) of the 26 families interviewed. It is important to note that forty (85%) cases of COVID-19 associated with the outbreak had never received any dose of COVID-19 vaccine; three persons (6%) were partially vaccinated having administered 1 dose of Moderna or Pfizer-BioNTech ≥14 days prior to a positive PCR test result but they had not received the second dose; four were (9%) were fully vaccinated because they have had 2 doses of Moderna or Pfizer-BioNTech or a single dose of Janssen (Johnson & Johnson, New Brunswick, NJ, USA) vaccine ≥14 days prior to a positive PCR test result.

These results suggest that the delta variant is highly transmissible in indoor sports settings and within families. This suggests multi-component prevention strategies, including vaccination, that remain important to reduce the spread of SARS-CoV-2, with a focus on people who play indoor sports † and their contacts [117].

The most heated debate concerns whether for COVID-19 there will be the same periodicity for new vaccinations as in the case of influenza with the difference that for the latter, despite being an infectious disease, vaccination is not mandatory. Undoubtedly, mRNA-based technology opens up new possibilities such as creating a vaccine that protects against most variants of SARS-CoV2. The biggest challenge appears to be to make enough changes to the mRNA platform vaccines to address the emerging variants. Pfizer and BioNTech have raised the possibility of administering a third dose of the vaccine BNT162b2 to increase immunological activity, to confer greater safety and efficacy against SARS-CoV-2 variants. One study was designed to make specific changes to BNT162b2 directed specifically against variant B.1.351 [117].

Modifications of mRNA-1273 with a booster dose for variant B.1.351 are under study [118]. In regard to the Novavax vaccine, the first generation of which has not yet been authorized in the United States, scientists are working on a booster dose or a bivalent combination vaccine, to increase the degree of protection from variants [119]. Recently, the 768 NVX-CoV2373 vaccine was efficacious in preventing severe COVID-19 disease due to the B.1.351 variant, resulting in mild to moderate manifestations of the disease due to the B.1.351 variant [119].

**Figure 3:**
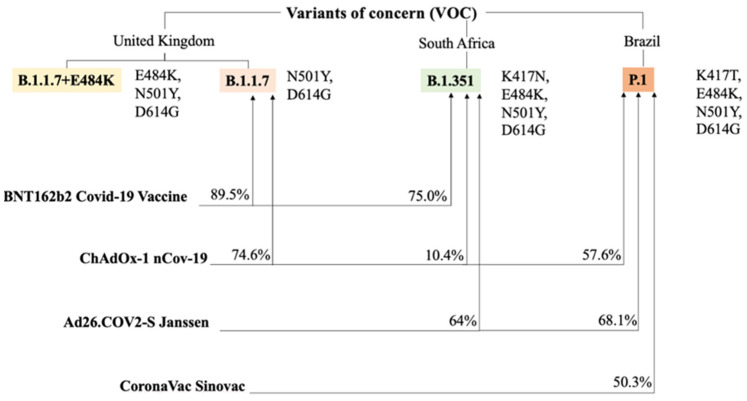
SARS-CoV-2 variants and the respective efficacy of most administered vaccines are shown [120]. B.1.1.7. i.e., the UK variant has recently been studied by investigators: 89.5% and 74.6% efficacies have been demonstrated against the variants by the B1.1.7 + E484K and ChadOx-1 nCoV-19 vaccines respectively.

**Figure 4:**
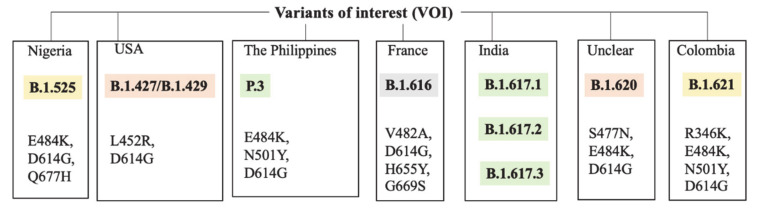
Variants of Interest (VOI) are illustrated. Every box reflects a country the first variant was discovered in. As for variants of concern, the Spike protein mutations associated with the variant are displayed. The efficacy of vaccines has not been reported due to the lack of data in terms of both literature studies and official national reports. Data are updated until 6 May 2021 [121].

The main challenge today lies in conducting studies comparing the degree of production of neutralizing antibodies against SARS-CoV2 elicited by prototype vaccines and engineered on the Wuhan-hu-1 variant from elicited antibody responses against the new variants of SARS-CoV2. In another study, scientists could use serum samples from people previously vaccinated with a prototype vaccine who are given an experimental booster dose against the more contagious variants (Figure 3 and Figure 4).

Percentages of efficacy from the other vaccines versus the main variants of concern (VOC) are also presented. Numbers mainly reflect efficacies against the symptomatic non-severe infection by SARS-CoV-2. Spike protein mutations are pictured on the side of each variant to which they belong [105].

### 3.7. SARS-CoV-2 Adenoviral Vector Vaccines and the Risk to Develop Thrombosis

SARS-CoV-2 vaccines were reported as safe and effective before their marketing and worldwide distribution by first, second, third phase clinical trials and pooled analyses.

In February 2021, large-scale epidemiological data started to raise suspicion of coagulopathies, after adenoviral vector-based vaccines reached millions of administered doses both in Europe and United States. In particular, the ChAdOx1 nCov-19, marketed by AstraZeneca and subsequently renamed Vaxzevria vaccine, has been considered responsible for the development of arterial and venous thromboembolism in a selected population, mostly of the female sex, under the age of 60 years [122].

A single dose (0.5 mL) of the vaccine has been formulated to contain about 2.5 × 10^8^ infectious units (Inf.U) of Chimpanzee Adenovirus encoding the SARS-CoV-2 Spike glycoprotein (ChAdOx1-S). The generation of the final product derives from the genetically modified human embryonic kidney (HEK) 293 cells. Recombinant DNA technology was also used for this intent [122].

Despite being rare events, around 1/100 000 recipients, further considerations are warranted and justified the European Medicines Agency (EMA) examination of those cases [123].

In April 2021, Greinacher et al. [124] reported 11 cases of thrombosis, including 10 multiple ones, involving cerebral and splanchnic veins with one death. Pulmonary emboli (PE) were also frequent in this subset of patients. Other conditions that required medical treatment included disseminated intravascular coagulation DIC and severe thrombocytopenia. The etiology has not been fully understood but it has become clear that anti-platelet factor 4 (PF4) antibodies in those patients’ serum were implicated and considered to have a fundamental role in what was initially described as similar to heparin-induced thrombocytopenia (HIT), with the difference being that heparin was not administered in all cases and antibody binding was successful even in the absence of heparin.

Under the assumption that immune mechanisms drove the syndrome, it was named VITT (vaccine-induced thrombotic thrombocytopenia). Of note is the time of development of the first symptoms, with an average of 1–2 weeks after the administration of the first dose. This is the reason why European governments started to reassure the population who already underwent the first dose of the ChAdOx1 nCov-19 vaccine to also receive the second one if no side effects were reported.

Schultz et al. [125] also published clinical cases of patients, mostly women, presenting with venous thrombosis, which included 2 sigmoid cerebral sinuses thromboses, a portal vein branch thrombosis, cerebral vein thrombosis, and a right cerebellar haemorrhagic infarction. In all of those cases, anti-PF4 antibodies were identified in patient plasma.

### 3.8. SARS-CoV-2 Vaccine-Induced Thrombotic Thrombocytopenia

Platelet factor 4 (PF4) is a platelet-derived cytokine of the CXC (chemokine) family. It is released by activated platelets to promote coagulation via neutralization of heparin-like molecules on endothelial cells. In concert with polyanionic proteoglycans (PGs) derived from endothelial cells, they create complex autoantibodies direct to these components. They have been identified by enzyme-linked immunosorbent assay (ELISA) and assays based on platelet activation, which, when tested, were enhanced by the addition of PF4. Thus, IgGs were found responsible for directly stimulating coagulation via FcγRIIA receptor-dependent mechanisms.

**Figure 5:**
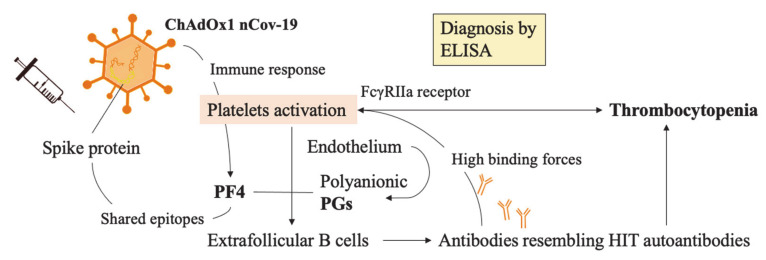
The proposed mechanism of autoantibodies generation is described. Following administration of adenoviral vector encoding the spike protein, a subsequent inflammatory cascade, stimulated by the individual immune response, activates platelets to generate platelet-factor 4 (PF4). In complexes with polyanionic PGs, derived from endothelial cells, they stimulate extrafollicular B cells to antibody production which, in turn, would exert positive feedback on platelet activation. The antibodies generated would resemble HIT autoantibodies from that point on with respect to thrombogenesis and hemostasis disorders. PF4 shares some epitopes with the Spike protein but, despite this, they are not sufficient to induce cross-reactivity. PF4: platelet factor 4, HIT: heparin-induced thrombocytopenia, PGs: proteoglycans.

Goldman et al. [120] recently proposed a model according to which the first activation of platelets may lead to PF4 release in the circulation. In turn, PF4 complexing with PGs stimulates extrafollicular B cells to produce anti-PF4 IgGs. From this point, the consequent molecular effects would then resemble heparin-induced thrombocytopenia (Figure 5).

Interestingly, Greinacher et al. have discarded the hypothesis of molecular mimicry mechanisms, due to the absence of cross-reaction with SARS-CoV-2 derived Spike protein [124]. The authors analyzed the Spike protein sequence and found 3 similar immunogenic epitopes, the part of an antigen molecule to which an antibody attaches itself with PF4. Prediction tools and 3D modeling software including IMED and SIM were used to compare them. Subsequently, they collected blood sera from 222 patients which tested positive for PCR analysis of SARS-CoV-2 infection and tested them for the presence of PF4/heparin ELISA, as well as heparin-dependent and PF4-dependent platelet activation assays [124]. Their results reinforce the hypothesis that the Spike protein is not inducing VITT.

Only 19 of 222 patients tested positive for PF4/heparin ELISA but those patients did not show any platelet hyperactivation sign. Furthermore, anti-PF4 and anti-PF4/heparin antibodies from two VITT patients were tested. They did not show any cross-reactivity to the recombinant SARS-CoV-2 spike protein [124].

The same research group 4 [121] had been studying the mechanisms underlying autoimmunity of HIT. By examining the binding forces expressed as pN (picoNewton) which are applied by antibodies in their binding to the target, they found substantial differences in terms of platelet activation. In particular, they divided groups of antibodies according to classes of force: group 1 showing pN < 60 antibodies (most of which do not activate platelets), group 2 with binding forces of 60 ≤ pN ≤ 100, which were found to activate platelets in the presence of polyanions, and group 3 with binding forces pN > 100, which bind to PF4 even without polyanions present. A statistical difference was recorded in the second (60 ≤ pN ≤ 100; *p* < 0.001) and third groups (pN ≥ 100; *p* = 0.006) [125]. Therefore, those higher forces were able to cluster PF4-molecules forming antigenic complexes which allow binding of polyanion-dependent anti-PF4/polyanion antibodies (anti-PF4/P-ABS). That induced massive platelet activation in the absence of heparin [121].

### 3.9. Updates from the European Medicines Agency

Since 7 May 2021, the PRAC committee has released more suspected reports of hemostatic and non-hemostatic conditions in other anti-COVID-19 vaccines under evaluation [126].

Myocarditis and pericarditis cases have been associated, even though a causative link has never been proven, with Comirnaty (the new brand name for Pfizer and BioNTech’s COVID-19 vaccine, BNT162b2). EMA has requested analyses of data from marketing authorization holders for both Comirnaty and Moderna [127]. Recent investigations include interesting cases of Guillain–Barre syndrome after vaccination with the Vaxzevria vaccine. Further detailed monitoring is necessary [127].

Some cases of thrombocytopenia have been reported for mRNA vaccines too, in particular Comirnaty and Moderna vaccines. Epidemiological studies are confirming the rate of those cases is lower than the general population incidence rate [128]. The BNT162b2 vaccine has also been the subject of EMA evaluations for cases of facial swelling in people with a history of injections with dermal fillers [127].

The Pharmacovigilance Risk Assessment Committee (PRAC) has decided to validate this adverse effect by inserting it in Cominarty’s product information. This was possible thanks to literature revision and the European database for suspected side effects EudraVigilance [127].

### 3.10. Public Health Challenges: Do Benefits Outweigh Risks?

Since the first cases of thrombosis for the Vaxzevria vaccine were reported [129], several studies tried to compare the incidence of those events with that of the general population. Pottegard and colleagues [128] assessed rates of hemostatic events in a cohort of 148,792 vaccinated Danes and 132,472 vaccinated Norwegians and compared them with general population cohorts from Denmark and Norway. Results showed 59 venous thromboembolic events in the vaccinated cohort compared with 30 expected based on general incidence rates. In particular, higher rates of cerebral venous thrombosis were observed: the standardized morbidity ratio, calculated as the ratio between observed and expected events, was demonstrated to be 20.25 (8.14 to 41.73). Interestingly, the rate of death was higher for the general population: 44 cases versus 15 for the vaccinated group [128]. However, it should be noted that the absolute risk of developing venous thromboembolic events was small (11 excess events/100,000 vaccines) and the results should be interpreted considering the proven beneficial effects of the vaccine. These data emerge clearly when the analysis was undertaken to investigate subgroup effects stratified according to gender and to the young versus middle-aged adults (age categories 18–44 years and 45–65 years). For example, when the analysis was restricted to women, no excess rate of thrombocytopenia/coagulation disorders was observed [128].

Increased surveillance could reinforce these findings and prove the beneficial effects of Vaxzevria vaccine outweigh the risks. Notably, the European Medicines Agency (EMA) started to investigate thrombotic events through the PRAC to review all conditions related to haemostatic alterations, (including thrombocytopenia and bleeding) after vaccines administration [127]. According to the pharmacovigilance legislation, additional monitoring is mentioned with a label on the package whenever it is needed. Furthermore, the medical literature is continuously monitored by EMA to guarantee suspect adverse reactions are correctly addressed. Thus, the status under which vaccines have been assigned by EMA is “conditional marketing, authorization granted”. This is true for Vaxzevria, Moderna, Janssen, as well as Pfizer and BioNTech’s Comirnaty vaccines [127].

### 3.11. Janssen Adenoviral Vector Vaccine and Cases of Thrombosis

The Ad26.COV2 vaccine has been associated with cases of cerebral venous sinus thrombosis (CVST), severe thrombocytopenia, and disseminated intravascular coagulation [129]. Muir et al. [130] described a patient who showed signs of autoimmune heparin-induced thrombocytopenia. The patient, a 48-year-old female, presented with general malaise and abdominal pain. From peripheral blood tests, thrombocytopenia with schistocytes on the blood smear was revealed. Coagulation tests also confirmed a low fibrinogen level, prolonged activated partial thromboplastin time, and an elevation in the D-dimer level. Factor V Leiden and Prothrombin G20210A gene mutation assessments were negative. Additionally, hepatitis, HIV, and lupus were tested for, but later ruled out. Investigators concluded the diagnostic workup with a computed tomographic (CT) scan of the abdomen and pelvis which demonstrated massive splanchnic venous thrombosis. After the patient developed a new-onset headache, a cerebral CT scan revealed cerebral venous sinus thrombosis involving the right transverse and straight sinuses.

The patient was further tested for anti-PF4-heparin antibodies which returned negative even though she was previously treated with unfractionated heparin. When evaluated for anti-PF4-polyanions antibodies, the patient tested positive (2.550 optical-density units [upper limit of the normal range], ≤0.399). She was therefore switched to therapy with argatroban. Thus, the case of thrombosis described by Muir et al. [130,131,132], demonstrates an update in literature reports associated with the Oxford-AstraZeneca vaccine [127]. Nevertheless, several authors, including Sadoff et al. [129], concluded that the Janssen vaccine and Vaxzevria, despite sharing some common features have different intrinsic structures.

Notably, the Ad26.COV2. S vaccine is composed of a human Ad26-based vector and Ad26 is from Ad species D. Its cellular receptor, is different from the Oxford-AstraZeneca vaccine which uses the Coxsackie and adenovirus receptor (CAR), CD46. The ChAdOx1 nCoV-19 vaccine is a chimpanzee adenovirus-based vector and Ad26 is from Ad species E. Thus, their biological characteristics are strongly different, even though the mechanism of immunologic response in the host is similar.

## 4. Conclusions

The SARS-CoV-2 infection has highlighted the importance of preventative medicine. The systemic effects of this are still relatively poorly understood, but several risk factors have been identified. The worldwide roll-out of vaccines has allowed the lifting of lockdown measures and reduced the spread of the disease. Ongoing epidemiological research is needed to monitor the variant strains and ascertain the ongoing efficacy of the vaccines against the virus.

## Figures and Tables

**Table 1:** Efficacies of COVID-19 vaccines are compared according to disease and infection prevention.

Anti-SARS-CoV-2 Vaccine Type	Efficacy at Preventing Disease (D614G and B.1.1.7.)	Efficacy at Preventing Infection (D614G and B.1.1.7.)
BNT162b2	91%	86%
mRNA-1273 (Moderna)	94%	85%
ChAdOx-1 nCov-2	75%	52%
Ad26.COV2-S (Janssen)	72%	72%
CoronaVac (Sinovac)	50%	43%
Sputnik V	92%	80%
Novavax	89%	77%
Sinopharm	73%	63%
Tianjin CanSino	66%	57%

Highlighted are efficacies exceeding the 75% of potency which we set as a point of comparison. Some variables are estimations available from literature studies (UK SIREN study). The general source is IHME—Institute for Health Metrics and Evaluation documents on data summaries. Data are updated until 26 April 2021 [105].

## Data Availability

Not applicable.
